# Sleep and protein synthesis-dependent synaptic plasticity: impacts of sleep loss and stress

**DOI:** 10.3389/fnbeh.2013.00224

**Published:** 2014-01-21

**Authors:** Janne Grønli, Jonathan Soulé, Clive R. Bramham

**Affiliations:** ^1^Department of Biological and Medical Psychology, University of BergenBergen, Norway; ^2^Norwegian Competence Center for Sleep Disorders, Haukeland University HospitalBergen, Norway; ^3^Department of Biomedicine and KG Jebsen Centre for Research on Neuropsychiatric Disorders, University of BergenBergen, Norway

**Keywords:** long-term potentiation, stress, sleep deprivation, mood disorder, gene expression, translation control, brain-derived neurotrophic factor, Arc/Arg3.1

## Abstract

Sleep has been ascribed a critical role in cognitive functioning. Several lines of evidence implicate sleep in the consolidation of synaptic plasticity and long-term memory. Stress disrupts sleep while impairing synaptic plasticity and cognitive performance. Here, we discuss evidence linking sleep to mechanisms of protein synthesis-dependent synaptic plasticity and synaptic scaling. We then consider how disruption of sleep by acute and chronic stress may impair these mechanisms and degrade sleep function.

## Introduction

Daily, we devote 6–9 h of our life to sleep, a physiological state marked by muscle relaxation and reduced responsiveness to our surroundings. Sleep is characterized by well-defined changes in brain activity as seen on the electroencephalogram (EEG). Sleep alternates between non-rapid-eye movement (NREM) sleep and rapid-eye movement (REM) sleep in a characteristic pattern known as the sleep cycle. In humans, the typical duration of one sleep cycle is approximately 90 min and one night typically consists of 4–5 sleep cycles. The architecture within the sleep cycles changes as the night progresses. Deep slow-wave sleep (SWS) predominates NREM sleep in the first half of the night, while the duration of REM sleep epochs progressively increases throughout the night. By the end of the night a REM sleep epoch may last for more than 30 min (Carskadon and Dement, 2011). Rodents have many, short-lasting (10–15 min) sleep cycles with REM-sleep epochs lasting from 30 s to 2 min (Vivaldi et al., 1994; Comte et al., 2006).

According to the two-process model for sleep regulation, sleep and wakefulness are driven by an interplay between circadian and homeostatic processes (Borbely, 1982). The circadian factor promotes sleep during certain periods of the day, and largely determines the timing and duration of the sleep period (Czeisler et al., 1980; Dijk and Czeisler, 1995). The homeostatic factor represents a sleep propensity that accumulates during time spent awake and is reflected by the amount and intensity of SWS (Borbely et al., 1981; Banks and Dinges, 2007; Riedner et al., 2007; Vyazovskiy et al., 2007). This biological drive for sleep may additionally be overridden and influenced by environmental and behavioral factors (e.g., voluntary awake, shift/night work, noise, caffeine intake).

Sleep is vital for human cognitive performance and health. However, over the last decades major societal changes have occurred that may impact sleep in a negative way. Some describe our modern life style as a “24-h society”. This refers primarily to an increased recourse to shift and night work and a prolonged use of electronic media that often delays bedtime, consequently altering both sleep duration and quality (Brunborg et al., 2011). Human and animal studies alike show that sleep restriction or sleep deprivation induces deficits in cognitive functions like behavioral alertness, performance, mood, and memory (Banks and Dinges, 2007; Rasch and Born, 2013). In parallel, advances have been made in elucidating sleep-dependent mechanisms at the cellular and molecular levels. Activity-dependent synaptic plasticity is considered essential for long-term adaptive changes in behavior, including learning and memory, and the regulation of mood and motivation. Understanding how synaptic efficacy and plasticity are modulated by sleep is therefore key to unlocking the specific contribution of sleep to cognition and the impact of sleep loss on cognition.

Here, we first outline and critically discuss current knowledge with regard to mechanisms and regulation of synaptic efficacy and long-term synaptic plasticity during sleep. Emphasis is placed on regulation of gene expression and protein translation important for the consolidation of persistent forms of plasticity. We then focus on the impact of acute and chronic stress on sleep quality and amount, and discuss how interactions between stress and sleep affect sleep-dependent gene expression, plasticity, and cognition.

## Synaptic plasticity and sleep

### Models of synaptic plasticity

A few hours of wakefulness or sleep can modify the molecular composition of excitatory synapses, change their efficacy and make synapses grow or shrink. Before discussing sleep-dependent regulation of long-term synaptic plasticity, it is opportune to review the major mechanisms by which synapses are strengthened or weakened, reshaped, and eventually stabilized at the molecular level.

Synaptic plasticity is the ability of a synapse to change in strength in response to use or disuse. Diverse forms of synaptic plasticity exist at excitatory, glutamatergic synapses in the mammalian brain. Among them are long-term potentiation (LTP), long-term depression (LTD), and homeostatic scaling of synaptic strength. LTP and LTD are sustained increases and decreases, respectively, in synaptic efficacy induced by patterned synaptic activity. Homeostatic scaling refers to the ability of a neuron to modulate its firing rate by globally increasing or decreasing synaptic efficacy on all inputs to the dendrite. In contrast to LTP and LTD, which are input-specific (Hebbian plasticity), homeostatic scaling does not affect the relative difference in strength between inputs (non-Hebbian plasticity).

Importantly, LTP and LTD share a common set of mechanisms even if they represent opposite changes in synaptic strength. Both forms of long-term synaptic plasticity require actin cytoskeletal remodeling within dendritic spines and changes in spine morphology. Many studies report enlargement of spines in LTP and shrinkage or loss of spines in LTD (Okamoto et al., 2004; Bourne and Harris, 2008). Similarly, both LTP and LTD require *de novo* protein synthesis, including local regulation of protein translation in dendrites (Bramham and Wells, 2007). Below, we outline some of the canonical mechanisms linked to LTP and LTD.

**Figure 1:**
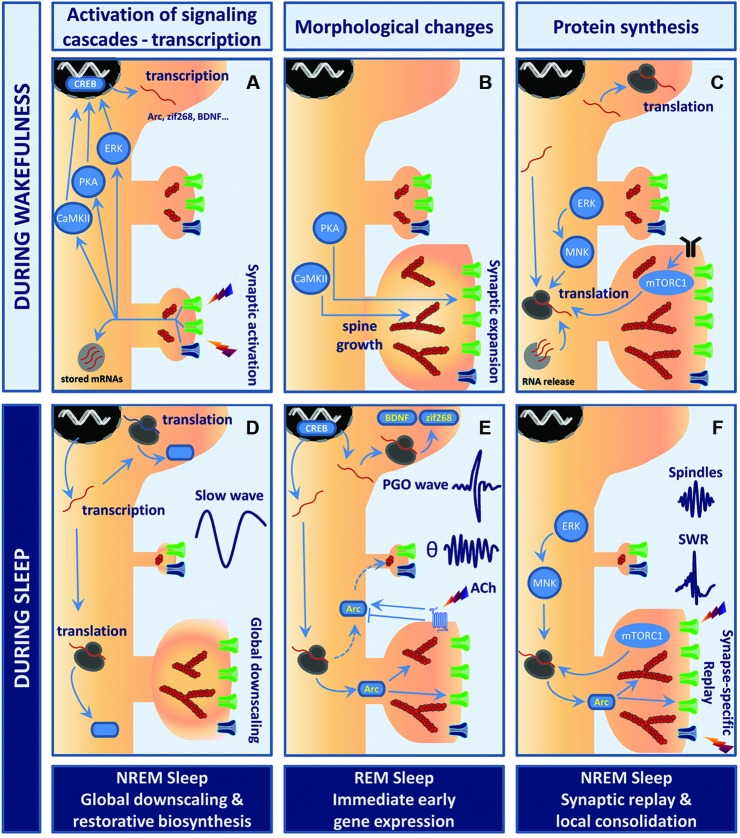
**Model of sleep stage-specific potentiation and homeostatic scaling.** In this working model, waking experience (LTP-like event) is consolidated through sleep stage-specific synaptic scaling, immediate early gene expression, and protein synthesis. Wakefulness **(A–C)**. **(A)** Stimulation of glutamatergic synapses leads to rapid calcium influx into the postsynaptic compartment via NMDAR (dark blue) and AMPAR (green). Elevation of calcium levels activates multiple kinases and signaling cascades (e.g., PKA, CaMKII, ERK) which converge toward transcription factors such as cyclic AMP response element-binding protein (CREB), thus triggering rapid immediate early gene (IEG) expression. **(B)** Within minutes, polymerization of actin into filaments (red, actin filaments (F-actin)) induces remodeling of the actin cytoskeleton within spines. While CaMKII likely contributes to bundling of F-actin and expansion of the actin scaffolds, PKA promotes the insertion of AMPARs into the postsynaptic membrane. This whole process results in robust growth of synapses and enhanced synaptic efficacy. **(C)** These changes are then wake-consolidated in a *de novo* protein synthesis-dependent manner. Newly transcribed IEGs are either translated in the cell soma or trafficked further into the dendrites to be processed by the local translation machinery. Neuronal activity may release and translate dendritically stored mRNAs (light gray circle). Both mammalian target of rapamycin complex 1 (mTORC1) and mitogen-activated protein kinase-interacting kinase (MNK) signaling enhance rates of translation initiation. Postsynaptic receptors depicted are TrkB (black), AMPAR (green) and NMDAR (blue). Sleep **(D–F)**. **(D)** NREM sleep supports the homeostatic process of cellular restoration by transcription and translation of genes involved in macromolecular biosynthesis and transport. In parallel, slow-wave activity (SWA) generates global synaptic downscaling in which synapses shrink and synaptic efficacy is reduced. Synapse-specific LTD at inactive or weakly active synapses may also be involved. **(E)** REM sleep and ponto-geniculo-occipital (PGO)-waves reactivate transcription of the plasticity-related IEGs Arc, brain-derived neurotrophic factor (BDNF) and zif268. Theta (θ) activity and increased acetylcholine levels regulate Arc protein turnover at the level of translation, degradation and mRNA decay. Arc may consolidate activity-induced synaptic changes by stabilizing the actin cytoskeleton and regulating trafficking of AMPAR from and to the postsynaptic membrane. **(F)** NREM sleep events like hippocampal sharp wave-ripples (SWRs) and thalamo-cortical sleep spindles have been suggested to actively take part in memory consolidation in conjunction with replay of neuronal activity patterns representing waking experience. The precise function of SWRs at the synaptic scale is yet to be unveiled. However, sparse and synapse-specific reactivations during SWRs of NREM sleep could provide bursts of local protein synthesis that consolidate synaptic modifications and memory formation. Thus, alternations of REM sleep-associated gene expression and NREM sleep-associated synaptic replay favor protein synthesis-dependent synaptic consolidation across sleep cycles.

LTP is divided into early (E-LTP) and late (L-LTP) phases which are mechanistically distinct. E-LTP typically lasts 1–2 h after LTP induction. This phase depends on the post-translational modification and trafficking of pre-existing proteins. It does not require new gene expression or protein synthesis. In brief, activated N-methyl-D-aspartate receptor (NMDAR)-type glutamate receptors trigger rapid entry of calcium into spines. Calcium influx impacts myriad signal transduction pathways, many of which are present within the postsynaptic spine itself. Initial signaling events include activation of numerous calcium-responsive protein kinases (calcium and calmodulin-dependent protein kinase II (CaMKII), extracellular signal-regulated protein kinase (ERK), and protein kinases A (PKA) and C (PKC) see Figure 1A). Activation of these pathways regulates both endosomal trafficking of AMPA receptors and modulation of actin cytoskeletal dynamics in spines, leading to enhanced postsynaptic membrane expression of AMPA-type glutamate receptors and a transient enlargement of dendritic spines (see Figure 1B; Malinow and Malenka, 2002; Bosch and Hayashi, 2012; Lisman et al., 2012).

The formation of stable L-LTP, which lasts many hours and days, requires new gene expression and protein synthesis (Stanton et al., 1984; Matthies et al., 1990; Costa-Mattioli et al., 2009; Sossin and Lacaille, 2010; Gal-Ben-Ari et al., 2012). The first period of protein synthesis occurs within the first 2 h following LTP induction. L-LTP is associated with stable enlargement and remodeling of the postsynaptic density (a large multi-protein complex attached to the membrane), enlargement of pre-existing dendritic spines, as well as *de novo* synapse formation (see Figure 1C; Lisman and Raghavachari, 2006; Bourne and Harris, 2008). Inhibition of protein synthesis prevents maintenance of the change in synaptic efficacy initiated during E-LTP. Additionally, protein synthesis inhibitors prevent stable increases in actin filaments (F-actin) associated with L-LTP without affecting actin cytoskeletal formation during E-LTP (Bourne et al., 2007; Bramham, 2008; Murakoshi and Yasuda, 2012).

LTD may be induced following activation of metabotropic glutamate receptors (mGluRs) and NMDARs. Unlike LTP, LTD expression relies on activation of phosphatases (i.e., PP1 and calcineurin) and is accompanied by removal of AMPARs from the postsynaptic membrane, thus lowering synaptic efficacy. Again, stabilization of the change in synaptic efficacy requires protein synthesis (but not necessarily new mRNA expression; Malenka and Bear, 2004). This is paralleled by a net decrease in spine F-actin and a shrinkage or retraction of dendritic spines (Tada and Sheng, 2006; Bosch and Hayashi, 2012).

Finally, it should be noted that LTP mechanisms probably differ between brain regions, and different input patterns can generate distinct forms of LTP (For detailed accounts see Ho et al., 2011; Panja and Bramham, 2014).

### Sleep loss impairs late long-term potentiation (LTP) and cognitive functioning

The impact of sleep loss on long-term synaptic plasticity has been investigated in recent decades. The majority of studies have employed sleep deprivation or sleep restriction to assess the benefits of sleep. Several protocols have been used to induce sleep loss.

#### Methods in sleep deprivation studies

The methods used in the vast majority of animal studies aim at disturbing sleep by total sleep deprivation, sleep restriction, or specific sleep stage deprivation. Common protocols include forced locomotion by placing the animal in a rotating drum, treadmill or platform, gentle handling (e.g., tactile, acoustic stimuli) and disturbance of the animal’s nesting material, or presentation of novel objects. Specific REM sleep deprivation is often achieved by placing animals on small platforms over water, the “flower-pot” technique. Differences in methodology may explain some discrepancies with regard to the effects of sleep loss on synaptic plasticity (Kopp et al., 2006; Vecsey et al., 2009; Havekes et al., 2012).

#### Sleep loss impairs long-term potentiation (LTP)

Several studies, mainly conducted in hippocampal tissue slices prepared from sleep-deprived rodents, have established that LTP expression depends on, or at least benefits from, a prolonged (non-disrupted) period of sleep. For instance, shorter (4–6 h) and longer (12–24 h) periods of sleep fragmentation by forced activity or total sleep deprivation by gentle handling impair LTP expression at Schaffer collateral-hippocampal subregion cornu ammonis 1 (CA1) synapses (Campbell et al., 2002; Kopp et al., 2006; Tartar et al., 2006; Vecsey et al., 2009). Importantly, 5–6 h of sleep deprivation specifically impairs LTP maintenance, leaving LTP induction intact (Vecsey et al., 2009; Florian et al., 2011). Investigating the bidirectionality (LTP/LTD) of synaptic modifications in CA1, Kopp et al. (2006) observed that 4 h sleep deprivation by gentle handling shifted the LTP/LTD induction threshold towards higher frequencies. Interestingly, this effect was reflected at the level of NMDAR subunit composition, suggesting that sleep deprivation modulates the function of postsynaptic membrane receptors supporting activity-dependent changes in synaptic efficacy (Kopp et al., 2006). However, a later study reported no effect on NMDAR function or LTP induction (Vecsey et al., 2009). The reasons for this discrepancy are unclear but could be related to differences in the sleep deprivation protocols. Kopp et al. (2006) employed 4 h of novel environment exposure, gentle knocking at the cage, and *ad libitum* access to nesting material, while Vecsey et al. (2009) used 5 h of gentle handling. Importantly, 2–3 min of daily acclimation handling does not disturb sleep or affect CA1 LTP (Vecsey et al., 2013).

The effect of sleep deprivation on LTD has not been studied in detail. One study indicates that 12 h of total sleep deprivation enhances expression of LTD induced by 20 Hz stimulation of the Schaffer collateral-CA1 region (Tadavarty et al., 2009). This particular stimulus paradigm evokes a generalized depression of synaptic inputs onto CA1 pyramidal cells (both activated and non-activated inputs are depressed; Sastry et al., 1984). There are no studies examining the effects of sleep deprivation on homosynaptic NMDAR-dependent LTD and mGluR-dependent LTD.

#### Loss of rapid-eye movement (REM) sleep impairs long-term potentiation (LTP)

Selective REM sleep deprivation produces deficits in hippocampal LTP similar to what has been observed after total sleep deprivation (both NREM and REM sleep). Prolonged REM sleep deprivation (24–72 h) impairs LTP in the hippocampus *in vitro* and *in vivo* (Davis et al., 2003b; McDermott et al., 2003, 2006; Ravassard et al., 2009; Alhaider et al., 2011). To assess whether REM sleep loss specifically impairs LTP maintenance in the dentate gyrus, rats were REM sleep-deprived for 4 h starting 1 h after LTP induction in wakefulness. LTP maintenance was reduced in REM sleep-deprived animals relative to control at 48 h (but not at 5 or 24 h). Total sleep deprivation similarly impaired L-LTP (Romcy-Pereira and Pavlides, 2004). Ishikawa et al. (2006) performed REM sleep deprivation for 24 h immediately after induction of LTP in the dentate gyrus of awake rats. LTP of the evoked population spike (which reflects synchronous neuronal firing) was strongly reduced compared to a non-sleep deprived group and yoked controls awoken in NREM sleep.

Importantly, REM sleep deprivation has opposite effects on LTP in different brain regions. In the study of Romcy-Pereira and Pavlides (2004), LTP maintenance in the medial prefrontal cortex was enhanced after 48 h of recording whereas LTP in the dentate gyrus returned to baseline levels after the same period (Romcy-Pereira and Pavlides, 2004). However, measurements of the population spike amplitude in the medial prefrontal cortex indicate increased neuronal excitability but not necessarily a change in synaptic efficacy. Taken together, current evidence suggests that sleep loss impairs the maintenance of LTP, at least in the CA1 and dentate gyrus regions of the hippocampus. The effects of sleep loss on cortical LTP have not been studied in detail.

#### Sleep loss impairs cognitive functioning

The impact of sleep loss on L-LTP is consistent with accumulating evidence regarding the benefits of sleep to cognitive functioning, including long-term memory formation (for a review, see Diekelmann and Born, 2010). Studies in rodents not only show that memory depends on sleep, but also that sleep must occur within a specific time window following learning. Indeed, mice subjected to 6 h sleep deprivation immediately after a complex object recognition task exhibit impaired memory retrieval, while memory is intact if sleep deprivation is performed 6 h after learning (Palchykova et al., 2006). A similar time-window is seen in contextual fear conditioning based on single-trial learning (Graves et al., 2003). Sleep deprivation from 0 to 5 h after conditioning impaired memory consolidation, whereas sleep deprivation from 5 to 10 h after training had no effect.

Earlier experiments in rats showed that REM sleep shortly after learning is necessary for the consolidation of memory. Selective REM sleep deprivation for 12 h immediately after or between 5–8 h after place (but not cue) learning in a Morris watermaze impaired long-term memory. REM sleep deprivation at other time points did not impair long-term memory formation. Notably, no impairment of place learning in the Morris watermaze occurred when REM sleep deprivation was applied 6 h after learning (Walsh et al., 2011). REM sleep periods increase in number and duration in active avoidance learning, and deprivation of post-trial REM during the period of enhanced REM sleep impairs long-term memory (Smith and Butler, 1982; Smith, 1996; Smith and Rose, 1996). These studies point to the existence of a time-window of REM sleep-dependent memory consolidation. To our knowledge, the possibility of a similar time-sensitive role for REM sleep in L-LTP maintenance remains to be explored.

Most studies in rodents have examined the role of total sleep or specific contributions of REM sleep in memory consolidation. In humans, specific functions for NREM and REM sleep have been proposed. While REM sleep mainly benefits the consolidation of procedural memories (skills), NREM sleep is implicated in consolidation of declarative and working memories (Gais and Born, 2004; Rasch and Born, 2013). Memory consolidation following tasks consisting of simple declarative material usually shows low susceptibility to REM sleep deprivation. In contrast, consolidation following tasks of higher complexity, or tasks which integrate procedural or emotional components, are more vulnerable to REM sleep deprivation (Rasch and Born, 2013). An interesting paradox originates from two studies where pharmacological (selective noradrenaline or serotonin re-uptake inhibitors) suppression of REM sleep enhanced, rather than impaired, memory consolidation (Vertes and Eastman, 2000; Rasch et al., 2009).

Overall, the evidence from rodents suggests that total sleep and REM sleep support cellular mechanisms that are used in the generation of stable LTP and long-term memory. As L-LTP and long-term memory depend on *de novo* gene transcription, it is important to consider how gene expression is regulated during normal sleep and sleep deprivation.

### Gene expression during sleep and after sleep loss

Studies on how sleep and sleep loss affect the regulation of gene expression have yielded insights into the molecular mechanisms at play during sleep. In addition, a handful of studies have explored sleep stage-specific regulation of gene expression.

The advent of genome-wide expression profiling (i.e., blood, brain tissue) allowing the screening of thousands of transcripts has given researchers the opportunity to look at specific effects of sleep vs. sleep loss in the brain (Cirelli et al., 2004; Mackiewicz et al., 2007). Sleep specific changes in the mouse cortex involve as many as 2090 mRNAs which increase or decrease in their steady-state expression (Mackiewicz et al., 2007). Most of the genes increased during sleep are linked to macromolecular biosynthesis and transport, supporting a restorative function of sleep at the cellular level. In healthy humans, just 1 week of insufficient sleep (6 h per day) affects the expression of 711 different mRNAs in whole blood relative to subjects getting sufficient sleep (8.5 h per day; Möller-Levet et al., 2013). Genes altered by sleep restriction in this study were involved in sleep homeostasis, circadian rhythms, oxidative stress, and metabolism. Moreover, the insufficient sleep was associated with poor cognitive performance in a vigilance test. These studies support the idea that biosynthetic pathways are “recharged” during sleep for optimal function during wakefulness. However, it is also possible that metabolic changes are required to support bursts of protein synthesis or other energy-expensive processes during sleep.

Only a few IEGs have been causally linked to late LTP and long-term memory. These genes include the activity-dependent cytoskeletal-associated protein (Arc, a.k.a Arg3.1), the transcription factor zif268 (a.k.a. egr-1, krox24, Ngfi-A), and the neurotrophin brain-derived neurotrophic factor (BDNF; Guzowski et al., 2000; Jones et al., 2001; Plath et al., 2006; Messaoudi et al., 2007; Bekinschtein et al., 2008; Penke et al., 2014). Given their essential role in consolidation mechanisms, these genes could be expected to be induced at one point or another during the NREM-REM sleep cycle.

#### Arc and zif268

Surprisingly, a few hours of sleep has been associated with decreased expression of various IEGs, including Arc and zif268, throughout the cerebral cortex (Cirelli and Tononi, 1999; Cirelli et al., 2004). Thompson et al. (2010) mapped Arc and zif268 expression, among other candidates, across mouse brain regions following sleep deprivation. Arc mRNA expression in the hippocampus and neocortex was higher during spontaneous wakefulness and after 6 h of sleep deprivation compared to time-matched sleeping controls. A similar pattern has been shown for Arc and zif268 mRNA after 8 h of sleep deprivation (Cirelli and Tononi, 2000).

Following bursts of synaptic activation, a fraction of the newly transcribed Arc mRNA is transported to dendritic processes for local storage, translation, or decay. Arc protein is implicated in LTP, LTD as well as homeostatic scaling (Bramham et al., 2010; Korb and Finkbeiner, 2011; Shepherd and Bear, 2011). In LTP, Arc functions to stabilize nascent F-actin (Lyford et al., 1995; Messaoudi et al., 2007). In LTD and scaling, Arc recruits the endocytic machinery (binds endophilin and dynamin) to facilitate endocytosis of synaptic AMPARs (Chowdhury et al., 2006; Rial Verde et al., 2006; Shepherd et al., 2006). zif268 regulates late response genes but a causal role for zif268-dependent gene expression in LTP or memory formation has not been established (Davis et al., 2003a; Knapska and Kaczmarek, 2004; Baumgärtel, 2009; Penke et al., 2014). Genetic or pharmacological (antisense oligodeoxynucleotide) inhibition of Arc and zif268 expression severely impairs long-term memory (Guzowski et al., 2000; Jones et al., 2001; Plath et al., 2006; Messaoudi et al., 2007).

Sleep disturbances may affect gene expression through decreased activation of several signal transduction pathways. Short sleep deprivation (5–6 h) reduces ERK and cAMP-PKA signaling (Guan et al., 2004; Vecsey et al., 2009). Both pathways regulate gene expression at the transcriptional level via CREB, a cAMP-responsive transcription factor. The transcription of Arc and zif268 underlying L-LTP is both ERK and cAMP/PKA-dependent (Davis et al., 2000; Waltereit et al., 2001; Ying et al., 2002; Kawashima et al., 2009). Mechanistically, Vecsey et al. (2009) showed that 5 h of sleep deprivation in mice enhances the expression of an enzyme that degrades cAMP, namely phosphodiesterase 4, thus reducing PKA activation. Five hours of sleep deprivation altered PKA signaling to CREB while impairing PKA-dependent forms of hippocampal LTP and long-term memory. Importantly, all these effects were rescued by treatment with phosphodiesterase inhibitors (Vecsey et al., 2009). Reduced activity in these signaling cascades might be predicted to dampen IEG expression, yet enhanced expression of many IEGs has been observed after sleep deprivation. The basis for the enhanced expression of IEGs is not clear but there is evidence for regional and gene-specific regulation. Arc mRNA is upregulated in the cortex and hippocampus (Thompson et al., 2010; Grønli et al., 2012; Vecsey et al., 2012), though expression of the plasticity-associated IEGs Homer1a and zif268 in the hippocampus is not changed by 5 h of sleep deprivation (Vecsey et al., 2012). High-resolution mapping of gene expression in the study by Thompson et al. (2010) also identified subcompartments of the cortex in which the IEG expression was decreased in sleep deprived mice.

Post-learning REM sleep is associated with enhanced expression of zif268 in the rat neocortex and hippocampus (Ribeiro et al., 1999, 2007; Ribeiro and Nicolelis, 2004). Remarkably, enhanced zif268 mRNA expression also occurs during REM sleep after LTP induction in the dentate gyrus (Ribeiro et al., 2002). This important finding shows that plastic changes evoked at a single afferent pathway during the waking state are sufficient to alter REM-linked gene expression. Romcy-Pereira et al. (2009) further identified several genes with enhanced hippocampal expression specific to post-stimulation REM sleep. The genes included a putative dendritically localized mRNA (J01878) and calcium/calmodulin-dependent protein kinase I (CaMKI), a protein involved in dendritic spine remodeling and calcium signaling in ERK-dependent LTP induction (Wayman et al., 2008).

Ponto-geniculo-occipital (PGO) waves are prominent phasic events of REM sleep, and recent evidence links increases in PGO wave activity to gene expression and memory formation (Mavanji and Datta, 2003; Ulloor and Datta, 2005; Datta et al., 2008). Following learning, an increase in PGO waves and enhanced expression of phospho-CREB, Arc and zif268 mRNA is found in the dorsal hippocampus and amygdala (Datta et al., 2008). Pharmacological suppression of PGO waves blocks the REM sleep-associated expression of these genes, while pharmacological activation of the PGO wave generator activates their expression. Taken together, these data implicate PGO waves as mediators of REM sleep-associated IEG induction of potential importance for the consolidation of synaptic plasticity during sleep.

NREM and REM sleep are associated with distinct neurotransmitter milieus which may differentially regulate gene expression (Brown et al., 2012). Cholinergic activity, in particular, is high during REM sleep compared to NREM sleep. Pharmacological stimulation, aimed at mimicking REM sleep-associated cholinergic activity, induced Arc expression in human neuroblastoma cells and somatodendritic expression of Arc protein in cultured rat hippocampal slices (Soulé et al., 2012). In neuroblastoma cells, Arc expression was controlled at the level of transcription, translation, proteosomal degradation, and mRNA decay. Although Arc is induced during REM sleep, nothing is known about the function of Arc in relation to LTP and LTD during sleep (see section “Working Model of Synaptic Plasticity Regulation During Sleep” for discussion).

#### Brain-derived neurotrophic factor

Expression of the secretory peptide, BDNF, is susceptible to sleep alterations. Unlike Arc and zif268, BDNF mRNA and protein are enhanced following sleep deprivation (of 8 and 48 h) in the rat hippocampus (Guzman-Marin et al., 2006).

BDNF is stored and released pre- and postsynaptically from glutamatergic synapses (Edelmann et al., 2014). BDNF activates TrkB receptors and induces protein synthesis-dependent LTP *in vitro* and *in vivo* (Kang and Schuman, 1996; Messaoudi et al., 2002, 2007; Panja and Bramham, 2014). In the dentate gyrus, BDNF triggers Arc expression on which L-LTP critically depends (Messaoudi et al., 2007). BDNF is rapidly transcribed following LTP induction (Castrén et al., 1993; Bramham et al., 1996; Wibrand et al., 2006), and BDNF protein synthesis is necessary for some forms of LTP (theta burst; Pang et al., 2004).

Sleep deprivation for 8 and 48 h decreases expression of several potential downstream gene targets of BDNF-TrkB signaling (Synapsin I, CREB, CaMKII, and BDNF itself) in the hippocampus, but not in the neocortex (Guzman-Marin et al., 2006). This suggests a differential susceptibility of these brain regions to sleep deprivation at the level of transcription and signaling. Consistent with the impact of sleep loss on both BDNF expression and maintenance of LTP, a recent study reports that 24 h of sleep deprivation abolishes the increase in BDNF expression associated with L-LTP in hippocampal region CA1 (Alhaider et al., 2011).

Waking exploratory behavior in rats is positively associated with cortical expression of BDNF as well as greater NREM slow-wave activity (SWA; Huber et al., 2007). Faraguna et al. (2008) show that local, unilateral cortical infusion of BDNF during wakefulness increases NREM SWA in the infused hemisphere without affecting REM sleep. Infusion of anti-BDNF antibody or K252a (which blocks TrkB kinase activity) during waking prevented the exploration-related increase in local SWA. The authors suggest that synaptic potentiation induced by local BDNF infusion results in local sleep regulation. In humans, a functional Val66Met polymorphism in the pro-BDNF gene causes impaired activity-dependent dendritic trafficking and secretion of mature BDNF protein. Supporting a role for BDNF in human sleep regulation, a recent study reported impaired intensity of SWA in NREM sleep in Val66Met carriers relative to Val/Val homozygotes under basal conditions and immediately following a 40 h period of waking (Bachmann et al., 2012). The Val/Met genotype is also associated with poorer performance in verbal working memory (Egan et al., 2003; Ninan, 2014).

To conclude, gene expression in the brain appears to be dynamically regulated across wakefulness and sleep. Microarray studies have identified families of genes implicated in metabolism, macromolecular biosynthesis and transport as important targets of state-transition. Plasticity-related IEGs such as Arc, zif268 and BDNF are also subject to differential transcriptional modulation during periods of sleep and sleep deprivation. Overall, sleep seems to downregulate IEG expression in the cerebral cortex, while stage (REM sleep)- and region-specific increases occur after learning and LTP induction. Such local and temporal modulations in gene expression may underlie variations in the direction, strength, and persistence of synaptic changes elicited during wakefulness by learning events.

### Protein synthesis, translation control, and sleep

A recent global quantification of gene expression in mammalian cells concludes that the cellular abundance of proteins is predominantly determined at the level of translation (Schwanhäusser et al., 2011). Translation proceeds in three phases: initiation, elongation, and termination. Translation initiation is the process whereby the mRNA is recruited to the ribosome. The translation factor, eukaryotic initiation factor 4E (eIF4E), is required for translation of most mRNAs. eIF4E binds to the 5′ terminal m^7^GpppN cap structure on mRNA and serves to recruit the scaffolding protein, eIF4G, and other factors to form the translation initiation complex. The critical interaction between eIF4E and eIF4G is regulated by eIF4E-binding proteins (4E-BPs). In its unphosphorylated state, 4E-BP is bound to eIF4E and translation is inhibited. Phosphorylation of 4E-BP catalyzed by the mammalian target of rapamycin complex 1 (mTORC1) triggers the release of 4E-BP and enhances translation (Gingras et al., 2001; Proud, 2007). ERK signaling to mitogen-activated protein kinase-interacting kinases (MNKs) is associated with enhancement of translation though the mechanisms are not fully understood. MNKs bind directly to eIF4G and catalyze the phosphorylation of eIF4E at Ser209. Phosphorylated eIF4E has decreased affinity for mRNA binding, which, in theory, could facilitate protein synthesis by recruiting initiation complexes and therefore more ribosomes to the RNA (Buxade et al., 2008). During translation elongation the polypeptide chain is formed as the ribosome moves along the mRNA. Eukaryotic elongation factor 2 (eEF2) plays a key role in catalyzing the translocation of peptidyl-tRNAs from the A-site to the P-site on the ribosome. When phosphorylated, eEF2 does not bind the ribosome and global translation is slowed down. By mechanisms that are not fully understood, translation of certain synaptic proteins (Arc, *α*CaMKII) is maintained or enhanced under conditions of eEF2 phosphorylation (Scheetz et al., 2000; Chotiner et al., 2003; Soulé et al., 2006; Park et al., 2008; Gal-Ben-Ari et al., 2012). eEF2 kinase, the only known kinase for eEF2, is regulated by calcium/calmodulin, mTORC1, and ERK signaling (Proud, 2007).

Several lines of evidence implicated sleep in the regulation of the protein synthesis. Two early studies involving *in vivo* incorporation of radioactive leucine in the brain revealed that global rates of protein synthesis were regulated during sleep (Ramm and Smith, 1990; Nakanishi et al., 1997). Both studies concluded that rates of protein synthesis correlate positively with the amount of NREM sleep. More recently, Vazquez et al. (2008) performed a proteomics screen of spontaneous sleep-wake state dependent changes in cortical protein expression and demonstrated rapid changes on the order of minutes. Sleep is associated with upregulation of numerous genes in the rodent cortex, including genes encoding translation initiation factors (eIF4b, eIF5, eIF3 subunits 3, 8 and 12), and eEF2 (Cirelli et al., 2004; Mackiewicz et al., 2007, 2008). Not surprisingly, proteomic studies indicate that changes in protein expression patterns depend on the duration of sleep deprivation. Short periods (6 h) of sleep deprivation altered expression of 11 proteins associated with synaptic function or cytoskeletal regulation in the basal forebrain cholinergic region (Basheer et al., 2005), while 7 days of sleep deprivation was associated with enhanced cortical expression of cytochrome C, the latter possibly indicative of metabolic stress (Cirelli et al., 2009).

In addition, a small subset of transcripts involved in tRNA activation is upregulated during sleep (Mackiewicz et al., 2007). Microarray analysis of mouse hippocampal tissue obtained after 5 h of sleep deprivation identified a decrease in the expression of mRNAs associated with protein translation (Vecsey et al., 2012). Independent validation confirmed decreased expression of total and phosphorylated mTOR following sleep deprivation. This difference was absent in mice permitted to sleep for 2.5 h after the sleep deprivation (rebound sleep). This upregulation of translation-related genes has been taken in support of active protein synthesis during sleep. Alternatively, translation factor synthesis in sleep may be restorative in nature, preparing the translational machinery for waking protein synthesis.

A recent study examined the relationship between sleep quality and quantity of home cage housed rats with the activity-state (phosphorylation) of translation factors eIF4E and eEF2 (Grønli et al., 2012). In the hippocampus, no association was found between sleep and translation factor activity. In the prefrontal cortex, more NREM sleep was associated with higher eIF4E and eEF2 phosphorylation. eEF2 phosphorylation correlated positively with sleep quality (total time spent in SWS) and negatively with poor sleep quantity (number of waking episodes). Levels of phosphorylated eIF4E correlated positively with the number of SWS and REM sleep episodes. Taken together, this suggests that sleep quality (based on the amount and number SWS episodes) correlates positively with phospho-eEF2 and phospho-eIF4E levels. These changes provide only an indirect measure of (enhanced) translational activity and more work is needed to profile the impact on translation and protein expression. However, dual eIF4E/eEF2 phosphorylation is mechanistically linked to protein synthesis-dependent forms of LTP in the dentate gyrus and plasticity (ODP) in the visual cortex (Kanhema et al., 2006; Panja et al., 2009; Seibt et al., 2012; Dumoulin et al., 2013).

Following 8 h of sleep deprivation, phosphorylation of eIF4E decreased in the dentate gyrus, but not in the CA region (Grønli et al., 2012). In contrast, eEF2 phosphorylation was elevated in both hippocampal regions and the prefrontal cortex. Thus, sleep deprivation has brain region-specific effects on translation initiation and elongation activity. Surprisingly, sleep deprivation increased Arc mRNA levels in the rat prefrontal cortex without affecting Arc protein expression. This dissociation between Arc mRNA and protein expression in sleep-deprived rats might be explained by enhanced ubiquitination and proteasomal degradation of Arc protein (Soulé et al., 2012). When Arc transcription is persistently stimulated, protein degradation imposes a powerful brake on protein expression.

Interestingly, sleep quality and quantity prior to sleep deprivation predicted the effects of sleep on translational factor activity in the prefrontal cortex, but not in the hippocampal regions. Phosphorylation of eEF2 was associated with previous SWS (positive) and waking episodes (negative), while levels of phosphorylated eIF4E were associated with prior episodes of SWS and REM sleep (positive). The implication may be that a good nights’ sleep prior to sleep loss diminishes the impact of sleep deprivation on protein synthesis (Grønli et al., 2012).

Direct evidence for protein synthesis-dependent consolidation of synaptic plasticity has come from studies of ODP in the cat visual cortex (Aton et al., 2009; Seibt et al., 2012; Seibt and Frank, 2012). Sleep consolidates ODP by strengthening cortical responses to non-deprived eye stimulation (Aton et al., 2009). In a recent study (Seibt et al., 2012), cats were given monocular deprivation for 6 h in either wakefulness or sleep, combined with 6 h of intracortical infusion of the mTORC1 inhibitor, rapamycin. Vehicle-infused controls exhibited enhanced phosphorylation of 4E-BP1 and enhanced cortical expression of Arc and BDNF (and other proteins). Rapamycin blocked the sleep-related protein expression and consolidation of ODP, but did not affect plasticity induced during wakefulness. Dumoulin et al. (2013) further showed that consolidation of ODP requires ERK-MNK signaling leading to eIF4E phosphorylation (Dumoulin et al., 2013), as was found during LTP consolidation in the dentate gyrus (Panja et al., 2009). Taken together these results suggest that mTORC1 and ERK-MNK signaling are both required for sleep-dependent protein synthesis and consolidation of ODP.

In sum, gene and protein expression during sleep is likely important for changes in synaptic efficacy and consolidation of waking experience during sleep. The next section discusses recent insights into how functional and structural plasticity are regulated during sleep.

### Synaptic efficacy and morphology during sleep

A decade ago, Tononi and Cirelli (2003) proposed a theory for a function of sleep in synaptic processes linked to cognitive functioning. Binding together a significant part of current knowledge of sleep, the synaptic homeostasis hypothesis highlights the role of sleep in the downscaling of synaptic strength after prolonged wakefulness. In this view, synaptic strengthening during wakefulness occurs via LTP-like mechanisms, while NREM SWA induces mechanisms of LTD or depotentiation throughout the cerebral cortex (Tononi and Cirelli, 2003, 2006, 2012). According to this model, SWA results in a global homeostatic downscaling of synaptic weights in which the synapses enlarged by LTP during wakefulness are reduced in size during sleep and the weakest synapses are eliminated. Such scaling may enhance signal-to-noise ratios for information encoded during waking. By preventing saturation of input strength, homeostatic downscaling may serve to retain the information encoding capacity of networks. In addition, morphological scaling of spines would offset the metabolic expense of maintaining large synapses.

Electrophysiological recording of miniature excitatory postsynaptic currents (mEPSCs; currents which reflect spontaneous release of neurotransmitters from single vesicles) of layer II/III pyramidal neurons in the frontal cortex of mice and rats demonstrates wake-related increases and sleep-related decreases in synaptic efficacy (Liu et al., 2010). The frequency and amplitude of mEPSCs was enhanced after the dark period (wakefulness) and decreased after the sleep period. Matching these electrophysiological changes, the abundance of GluA1-containing AMPARs in biochemically fractionated synaptosomes was 40% higher after wakefulness than after sleep (Vyazovskiy et al., 2008; Hinard et al., 2012). Dephosphorylation of GluA1 on Ser845 is associated with decreases in channel open probability and decreased surface expression of AMPARs. Hinard et al. (2012) show that Ser845 phosphorylation is enhanced according to time spent awake, which appears compatible with the lack of synaptic depression at the level of AMPAR regulation during wakefulness. The fact that these changes are detected in synaptosomes from whole cortex and hippocampus is consistent with global scaling at the synaptic level.

Two recent studies provided evidence for distinct roles for NREM and REM sleep in modulation of synaptic plasticity. Chauvette et al. (2012) measured local field potentials in the rat somatosensory cortex of head-restrained cats during wake, comparing responses obtained before (wake 1) and immediately after (wake 2) a period of NREM. The responses were enhanced in wake 2, and longer periods of NREM were associated with larger evoked responses. A large transient increase in the response was observed in wake 2 but not after additional periods of NREM or REM sleep. The fact that delta power was increased in wake 2 compared to wake 1 is indicative of sleep inertia (“sleepiness”). Hence, it is possible that state-dependent modulation of synaptic efficacy contributes to the transient enhancement of the response (Winson and Abzug, 1978; Bramham and Srebro, 1989). However, a smaller stable increase in the evoked response was present in wakefulness after several sleep cycles, and this stable increase was mimicked by *in vitro* stimulation and intracellular hyperpolarization designed to mimic cortical slow wave “downstates” of NREM sleep (Chauvette et al., 2012). In sum, the work suggests that rapid upscaling (potentiation of the evoked responses) can occur in a cortical network during NREM sleep.

Grosmark et al. (2012) reported a prominent role of REM sleep in sleep-related neuronal plasticity. They show that overall firing rates of hippocampal pyramidal cells and interneurons increase moderately during NREM sleep periods, but decrease more during REM sleep, giving an overall net decrease in global firing from the neuronal population across a sleep cycle. Of major significance is the observed difference in pyramidal neuron firing during and between the intermittently occurring phasic events of NREM sleep known as sharp wave-ripples (SWRs). SWRs are irregular, synchronized bursts of neuronal activity in the hippocampus which are synchronized with thalamo-cortical spindle activity (Buzsáki et al., 2013). Grosmark et al. (2012) observed overall firing decreases in the periods between SWRs, but the synchrony and mean firing rate during ripple events increased across sleep in correlation with the power of the REM sleep theta rhythm. These findings are compatible with global downscaling of neuronal firing between SWRs and upscaling during SWRs. It is currently unclear whether these changes in neuronal firing and synchrony are mediated by LTP/LTD-type events on hippocampal pyramidal and interneurons. For further discussion see Born and Feld (2012), Tononi and Cirelli (2012), Cirelli (2013), Frank (2013), and Rasch and Born (2013).

At the anatomical level, two-photon microscopy has been used to visualize changes in dendritic spines of cortical neurons during sleep. Maret et al. (2011) showed that wakefulness is associated with a net increase in dendritic spines while sleep is associated with net spine loss. Yang and Gan (2012) ascribed the loss of spines during sleep to higher rates of turnover. However, it is not known whether spines size and density varies in a sleep-stage specific manner as shown for synaptic field potentials and neuronal firing activity.

### Working model of synaptic plasticity regulation during sleep

The evidence reviewed suggests that sleep-stage specific changes in synaptic efficacy and plasticity, firing activity, and network synchrony develop over the course of sleep. However, no consensus exists on how synaptic efficacy is regulated across the sleep cycle. In Figure 1, we offer a scenario for how synaptic plasticity is regulated during sleep. The working hypothesis is an attempt to integrate the cell biology of synaptic plasticity with the electrophysiological data.

We propose that specific cell biological events underlying homeostatic scaling and synaptic potentiation are parsed to specific stages of sleep and stage-specific population events. Downscaling during NREM sleep is supported by electrophysiological, biochemical and morphological data (Figure 1D; Grosmark et al., 2012; Cirelli, 2013). During REM sleep, immediate early genes such as Arc and zif268 are triggered by PGO-waves (Figure 1E; Ribeiro et al., 2002; Ulloor and Datta, 2005). Pharmacological cholinergic activity mimicking phasic REM sleep epoch also drives Arc expression in glutamatergic neurons (Soulé et al., 2012). Hence one function of REM sleep in this model is to provide immediate early gene induction in a broad population of cortical and hippocampal project neurons. A large body of work suggests that neuronal ensemble activity representing recent learning during the wake state is replayed in a time-compressed format during the SWRs of NREM sleep (Lee and Wilson, 2002; Skaggs et al., 2007; O’Neill et al., 2010). Grosmark et al. (2012) showed that firing synchrony during SWRs develops gradually over successive NREM-REM sleep cycles. It follows that sparse, but synchronous synaptic firing, is repetitively replayed during NREM SWRs (Figure 1F). Thus, in NREM sleep an interplay may exist between synaptic potentiation and homeostatic scaling, with synaptic potentiation occurring during SWRs and scaling during the inter-ripple periods. Restorative macromolecular synthesis of the translational machinery (ribosomal proteins, translation factors, tRNA) occurs during sleep and may function to support bursts of synaptic protein synthesis.

As a multifunctional dendritically translated protein, Arc could play a role in coordinating diverse forms of plasticity during sleep. In REM sleep, Arc mRNA would be synthesized and transported to dendritic processes (Figure 1E). During NREM sleep, the synaptic activity of SWR events is proposed to drive local translation of Arc and other dendritically localized mRNAs. Repetitive bursts of translation during the night would ensure synapse-specific, protein synthesis-dependent potentiation (Figure 1F). Extrapolating from LTP studies, local Arc synthesis would consolidate synaptic potentiation through regulation of actin cytoskeletal dynamics and enlargement of dendritic spines (Fukazawa et al., 2003; Messaoudi et al., 2007). The extremely rapid rates of Arc mRNA and protein degradation are well-suited for mediating bursts of protein expression during SWRs. Arc mRNA is subject to rapid translation-dependent decay (perhaps limiting synthesis to translation by a single ribosome), while Arc protein is rapidly ubiquitinated and targeted for degradation in the proteasome (Rao et al., 2006; Giorgi et al., 2007; Soulé et al., 2012). In the same neurons, Arc protein could function to mediate homeostatic scaling and LTD. Dendrite-wide downscaling might be achieved through nuclear import of Arc leading to downregulation of GluA1 transcription (Korb et al., 2013), or through selective targeting of Arc to inactive or weakly activated synapses resulting in Arc-dependent endocytosis of AMPARs (Shepherd et al., 2006; Beique et al., 2010; Okuno et al., 2012). Clearly, it will be important to elucidate the time-dependent functions of Arc, and the possible role of post-translational modifications of the protein in dictating its localization and function (Bramham et al., 2010; Craig et al., 2012).

As summarized in the above sections, sleep loss can actively affect synaptic plasticity, synaptic efficacy and cognitive functioning. Such outcomes are sensitive to the various protocols used to induce sleep loss. Enforcing wakefulness when the brain is programmed to sleep may induce effects unrelated to sleep loss per se. Sleep restriction or sleep loss are often associated with an increase (but temporary) in the activity of the neuroendocrine stress systems by altering the state or function of the hypothalamo-pituitary-adrenal (HPA) axis. Most of the studies investigating the impacts of sleep loss on synaptic plasticity are performed in brain regions sensitive to stress. Among them is the hippocampus, which is involved in the negative feedback response to stress and helps to determine whether stress is ongoing. Since sleep deprivation can be stressful, it is important that studies aim to control for such non-specific effects. Several studies discussed in this review controlled for hormonal stress response following sleep deprivation (Palchykova et al., 2006; Hagewoud et al., 2010; Süer et al., 2011; Grønli et al., 2012), signifying that sleep loss rather than stress perturbs the changes in synaptic plasticity. From this emerges the question of how stress impacts sleep and synaptic plasticity, to which we now turn our attention.

## Impact of stress on sleep (and synaptic plasticity)

Many people report that they feel stress due to perceived demands that exceed their resources. Our modern “24-h society” is one of several environmental stressors which disturb sleep. On work days we sleep about 38 min less than we did only a decade ago (Roenneberg, 2013). Stress is inevitable and has many (positive and negative) effects on the central nervous system. Stress is a perceived situation or experience which requires immediate compensatory responses for the maintenance of homeostasis. Importantly, if controllable, the body will adapt to stress, induce a fast energy input and improve cognitive achievements.

Stressful stimuli release stress hormones (glucocorticoids; cortisol in humans and corticosterone in rodents) that may have beneficial or detrimental physiological effects. Glucocorticoid receptors are widely expressed throughout the body and have a particularly dense distribution in the brain (De Kloet et al., 2005). Basal levels of corticosterone support LTP expression in hippocampus, whereas higher levels, stress or exposure to a new environment favor LTD (Pavlides et al., 1996).

Stress itself often disturbs sleep. Moreover, experiencing sleep loss following stress exposure may further potentiate changes in brain functioning at the level of synaptic plasticity. Vice versa, stress exposure after sleep loss alters the HPA response. In rats, the HPA response to restraint stress is reduced after 48 h sleep deprivation and 8 days of restricted sleep (but not after 1 day; Meerlo et al., 2002). In humans, partial (04–08 a.m.) and total (11 p.m. to 08 a.m.) sleep loss increases cortisol levels and delays the recovery of the cortisol release from the HPA axis (Leproult et al., 1997). Hence, sleep loss may affect the resilience of the stress response and potentiate the cognitive consequences of glucocorticoid excess.

The various paradigms employed in stress research rely either on acute or chronic, predictable or unpredictable stress. At present, little is known regarding the synaptic effects of stress-sleep relationship. However, it is expected that disturbed sleep and/or synaptic plasticity resulting from stress or manipulations of stress hormones depends on the intensity and duration of the treatment. Few studies have addressed this so far and no conclusion can yet be made. In the next sections we point to evidence that stress, either acute or chronic, predictable (controllable) or unpredictable (uncontrollable), can influence sleep and synaptic plasticity, but differently.

### Acute stress, sleep and synaptic plasticity

Acute stress like social defeat, tail suspension, restraint, forced swim, or foot shock are developed as tools to mimic an immediate threat resulting in despair-like behavior. The behavioral effects (e.g., sleep changes) are often transient, typically gone within 1–3 days after termination of the stressor (Meerlo et al., 1997; Kinn et al., 2008).

Exposure to social defeat induces changes in NREM sleep but leaves REM sleep unaffected. An immediate increase in deep NREM sleep which dissipates during the following 12 h has been reported (Meerlo et al., 1997). Such an increase re-occurs 4 days after defeat and dissipates again within 14 days after social defeat (Kinn et al., 2008; Kinn Rød et al., 2014, in press). Inescapable foot-shock has been shown to increase wakefulness and then decrease REM sleep (Sanford et al., 2010; O’Malley et al., 2013). Additionally, Philbert et al. (2011) report long-lasting increases in sleep fragmentation (21 days after the stress exposure; Philbert et al., 2011). Similarly, wakefulness is also reported to increase after 1–2 h of restraint or forced swimming, while REM sleep increases during the sleep rebound (Cespuglio et al., 1995; Dewasmes et al., 2004). Hence, both NREM and REM sleep are differently affected by the nature of the stressor.

To the best of our knowledge, no study has directly focused on the acute stress–sleep interaction at the synaptic level. Studies of stress alone show that restraint suppresses maintenance of hippocampal LTP, enhances LTD *in vitro* and *in vivo*, and impairs cognitive functions like spatial memory (Foy et al., 1987; Bodnoff et al., 1995; Kim et al., 1996; Xu et al., 1997; Conrad et al., 2004; Krugers et al., 2006; Chen et al., 2010). Foot shock also facilitates LTD induction and slightly impairs learning of a spatial task directly after stress exposure, but enhances memory retrieval 5 days later (Xiong et al., 2003). Despite the transient sleep changes reported to occur after social defeat, this stressor is shown to produce long-lasting effects on hippocampal LTP and LTD. Artola et al. (2006) showed that the threshold for LTP induction is still raised and that for LTD lowered 7–9 months after defeat and individual housing (Artola et al., 2006).

Following a short stressful experience, *de novo* gene expression and protein synthesis, which are crucial to long-term synaptic changes, are rapidly but transiently altered. Activity of the ERK pathway and its downstream targets (including zif268) are increased after restraint and forced swimming (Gutièrrez-Mecinas et al., 2011), and ERK activation mediates the effects of restraint tail shock stress on hippocampal LTP (Yang et al., 2004). Other IEGs necessary for the stabilization of activity-dependent synaptic plasticity are also affected by acute stress. Arc and BDNF are among those and their expression varies in a region-specific manner. Cortical upregulation of both Arc mRNA and protein expression is indeed detected after restraint (Mikkelsen and Larsen, 2006). Restraint also induces a rapid, transient modification of BDNF expression across several brain regions. Importantly, the impact of stress depends on the individual’s age. Defeat during adolescence and adulthood differentially regulates expression of several plasticity-related IEGs. A recent study shows that mRNA levels for Arc and BDNF (among others) are elevated following social defeat in adolescence, but not in adulthood (Coppens et al., 2011).

Compiled evidence suggests that acute stressful events have the capacity to induce sleep disturbances and alter long-term synaptic plasticity. Unfortunately, there is no data available on the effects of stress-sleep interactions on LTP, LTD, *de novo* gene expression or protein synthesis after acute stress.

### Chronic stress, sleep and synaptic plasticity

When stress becomes chronic the physiological changes are more profound and long-lasting. Importantly, the changes vary accordingly with the intensity, frequency, and particularly the unpredictability of the stressor. The unpredictability is of importance to overcome stress habituation that occurs if the stressors are given repeatedly in a controllable manner.

The various protocols of repeated stress have been shown to affect sleep differently. Animals remain awake throughout a 6 h recording period after exposure to 2 days of inescapable foot shock; in contrast, 3–5 days exposure decreases wakefulness and REM sleep (Papale et al., 2005; O’Malley et al., 2013). Four days of forced swimming or restraint both decrease NREM sleep, and restraint additionally decreases REM sleep compared to baseline (Papale et al., 2005).

Repeated exposure to stressors may constitute an environmental risk factor for the development of anxiety and depression. When restraint is given repeatedly, 2 h for 10 days in rats, REM sleep is altered for at least 21 days after termination of the stressor (Hegde et al., 2011). Importantly, the impact of restraint stress on REM sleep was bimodally distributed. One group of rats manifested an increase in REM sleep and anxiety-like behavior, while the other group showed reduced REM sleep and no anxiety-like behavior (Hegde et al., 2011). One animal model that was developed to mimic minor daily hassles is chronic mild stress (Willner, 2005). Various mild stressors unpredictably given for 4 weeks decrease deep NREM sleep and increase time in REM sleep and wakefulness (Cheeta et al., 1997; Grønli et al., 2004, 2012). These sleep changes parallel those found in human depression. Although sleep alterations are one of the hallmark symptoms of depression and anxiety, there is limited research in rodents on the role of sleep in stress related depression and anxiety. Further research on sleep, depression- and anxiety related behaviors is an interesting direction for future investigation.

Hippocampal LTP maintenance is suppressed, and LTD enhanced *in vitro* and *in vivo* after acute and chronic restraint. This is also found when restraint is given in combination with tail shock as well as chronic corticosterone or chronic stress exposure (Foy et al., 1987; Bodnoff et al., 1995; Kim et al., 1996; Xu et al., 1997; Krugers et al., 2006; Chen et al., 2010). Brief exposure to mild stress also affects synaptic plasticity, as induction of LTP is blocked and LTD is facilitated (Xu et al., 1997). Moreover, this facilitation of LTD is abolished by acclimatization to, or removal from the mild stressors (Xu et al., 1997).

Chronic stress, in general, is associated with changes at the transcriptional and translational levels. At the transcriptional level, chronic stress has been shown to both increase (defeat and novel cage; Pardon et al., 2005) and impair CREB activity (glucocorticoid treatment; Föcking et al., 2003, chronic mild stress; Grønli et al., 2006). These contrasting results may relate to differences in glucocorticoid concentration release due to different intensity or chronicity of the stressor. At the translational level, chronic stress enhances phosphorylation of the translational regulators eIF4E and eEF2 in prefrontal cortex but not in the hippocampus or dentate gyrus (Grønli et al., 2012). This upregulation of translational activity may be taken as evidence in support for active protein synthesis after stress in the cortical areas.

Recently, sleep-stress interaction was examined at the translational level using the chronic mild stress model. Being chronically stressed abolishes associations between an individuals’ sleep quality/quantity and translational activity. The sleep parameters are no longer predictive for cortical activity of initiation factor eIF4E and elongation factor eEF2 (Grønli et al., 2012). Similarly, chronic stress abolishes associations between sleep parameters measured *prior to* 8 h sleep deprivation and cortical translational activity as assessed after sleep deprivation. Given that persons experiencing chronic stress and depressed patients complain of non-restorative sleep, it is tempting to speculate that such lack of association between sleep quality and optimal rates of protein synthesis may be one of the underlying causes. Moreover, the effect of 8 h of sleep loss is modulated after chronic stress. In stressed rats, decreased activity of cortical eEF2 was found, whereas increased eEF2 activity occurs in non-stressed animals. No change of these translational regulators was observed in hippocampus. This may suggest that sleep deprivation counteracts the effect of chronic stress on eEF2 activity, in a region-specific manner. Interestingly, acute sleep deprivation has been reported to have antidepressant effects in humans (Wu and Bunney, 1990). The findings from animal studies raise the possibility that sleep deprivation may serve to restore or optimize rates of cortical protein synthesis in depressed patients.

Recent work shows that circadian changes in glucocorticoids are necessary for the formation and stabilization of dendritic spines in cortex after motor learning, and chronic and excessive exposure to glucocorticoids destabilizes learning-associated spines and impairs memory retention (Liston et al., 2013).

### Early life stress, sleep and synaptic plasticity

The brain is in constant change across the lifespan, starting from the early stages of life in utero. Early life (pre- and postnatal, as well as childhood and adolescence) hosts important developmental phases which allows the brain to mature. Being exposed to early life stress such as prenatal stress, maternal separation, low maternal care, or stress during adolescence has consistently been found to alter stress sensitivity in adulthood (Lupien et al., 2009).

Mammals show large amounts of active sleep (that parallels adult REM sleep) during early postnatal brain development. The predominance of REM sleep during early life is often taken in support of a role for REM sleep in processes of brain maturation and plasticity (Frank, 2011). The studies on sleep-related changes are scarce and the findings are divergent. Exposure of stress in utero may result in a prolonged first REM sleep episode and less NREM sleep in adulthood, compared to non-stressed controls (Rao et al., 1999). Long maternal separation (typically 3 h per day in the first 2 postnatal weeks) is reported to diminish the quality of deep NREM sleep, to alter total sleep time (decrease or increase), and to increase wakefulness compared to non-handled, handled, and brief maternally separated offspring. Moreover, the negative feedback regulation of the HPA axis in long maternal-separated offspring is suggested to be impaired and corticosterone level is elevated in long compared to brief maternal-separated offspring (Mrdalj et al., 2013).

Altered stress sensitivity in adulthood is also reflected in sleep changes. Exposure to later life stressor(s) affects sleep differently according to early life experience. Adult exposure to acute stress (2 h of cold) is followed by decreased REM sleep and elevated corticosterone levels, both in long maternal-separated offspring and handled controls (Tiba et al., 2004). Adult experience of chronic unpredictable mild stressors induces more time in sleep, more REM sleep episodes and more NREM sleep episodes ending in REM sleep in long, compared to brief, maternally separated offspring (Mrdalj et al., 2013). REM sleep deprivation in adult long maternal-separated offspring seems not to potentiate a present memory deficit (Garcia et al., 2013).

Independently of wakefulness, NREM or REM sleep, early life stress reduces brain activity measured by EEG, an effect potentiated by exposure to chronic stress as adults (Mrdalj et al., 2013). Offspring that receive low maternal care show poor LTP when they are adult, as opposed to those that were given high maternal care (Champagne et al., 2008). Single (short and prolonged), or repeated maternal separation can affect LTP expression in hippocampus and prefrontal cortex (Cao et al., 2013), without any change in the number of neurons and astrocytes (Baudin et al., 2012).

Activation of synaptic plasticity-related genes is assumed to represent an early step in the adaptation of neuronal networks to a stressful environment. Maternal separation after the first 2 postnatal weeks, at day 14–16, induces rapid increase in hippocampal Arc and zif268 mRNAs, accompanied by morphological changes such as an increase in spine number on CA3 dendrites (Xie et al., 2013). In rats exposed to isolation rearing, cortical upregulation of Arc mRNA and increase in both Arc and BDNF proteins is observed (Wall et al., 2012). Note that single (short and prolonged), or repeated maternal separation alter hippocampal BDNF expression (Roceri et al., 2002, 2004; Koo et al., 2003; Fumagalli et al., 2004; Nair et al., 2007). Notably, the prior history of maternal separation impacts the effect of adult stress on BDNF transcripts via modulation the upstream transcriptional activator CREB. An impact on neuronal progenitor proliferation is also reported, suggesting that alterations in CREB/BDNF may contribute to individual differences in hippocampal networks (Nair et al., 2007). In the cortex, the length of early life manipulations appears to be more important for these changes than their timing. Repeated early life stress induces a clear reduction of cortical BDNF levels in adult animals (Koo et al., 2003; Fumagalli et al., 2004; Roceri et al., 2004), whereas a single maternal deprivation does change BDNF expression (Roceri et al., 2002).

Stressful events early in life induce long-term sleep disturbances and alter long-term synaptic plasticity. Unfortunately, as for acute stress, there is no available data regarding the effects of stress-sleep interactions on LTP, LTD, *de novo* gene expression or protein synthesis after early life stress.

### Factors important for stress-sleep interactions

The findings on sleep changes after stress discussed above, raise an important issue that different stress modalities result in distinct sleep responses. Moreover, stress responses are mediated through the concerted activity of many brain areas and induce structural changes in neuronal networks. Changes can be short or long-lasting (Fuchs et al., 2006).

Brain areas involved in the stress response include areas important for sleep and wakefulness; the hypothalamus (including deep NREM sleep active neurons in the ventral lateral preoptic area), amygdala (activity is depotentiated during REM sleep), hippocampus (generating theta activity in REM sleep), prefrontal cortex (generating the highest voltage and the slowest NREM sleep waves compared to other cortical regions) and numerous brainstem regions promoting wakefulness like the locus coeruleus and raphe. Stress-induced changes in the activity of one or several of these brain regions may explain the different sleep changes.

The recovery from sleep loss is sensitive to stress. Likewise, recovery from stress is sensitive to sleep disturbances. If the individual has been exposed to stress prior to the sleep loss, the sleep recovery may be altered. Little data is available on how sleep recovery is affected by stress experience prior to sleep loss. One study has shown that exposure of rats to social defeat prior to 6 h of sleep deprivation potentiated changes in the recovery sleep by an increase in deep NREM sleep (Meerlo et al., 2001).

The available data on sleep disturbances and drive for sleep after acute stress suggests that stress accelerates the buildup of sleep need. NREM and REM sleep are differently affected by the nature of the stressor. Restraint increases REM sleep while social defeat increases deep NREM sleep. Cognitive functioning is considered to be potentiated and LTP-like changes facilitated after transient stress (Luine et al., 1996; Shors, 2001). However, more studies are needed to define the selective role of NREM and REM sleep rebound after stress. Moreover, knowledge on how prior stress may impair the sleep recovery after sleep loss is limited.

The recovery of stress may result in a long-lasting disruption of normal circadian sleep pattern by decreased or increased sleep throughout the 24 h period. Again, changes in specific sleep stage depend on the type of stressor. In the rats’ *active* phase restraint decreases sleep efficiency, NREM and REM sleep, whereas foot shock, swimming, cold as well as chronic controllable stress increase REM sleep 4 days after the stress exposure (Kant et al., 1995; Papale et al., 2005).

Behavioral factors appear to be important for the understanding of the variations in sleep changes brought by stress. During a stress situation, the coping strategy may play a significant role. Animals fighting back during a social conflict before being defeated show fragmented NREM sleep, an effect becoming more robust in the long-term (day 21 post defeat) compared to animals showing quick submission and passivity (Kinn Rød et al., 2014, in press). Importantly, increases in REM sleep have been observed if the organism controls the stressor (e.g., escapable foot shock; Kant et al., 1995; Sanford et al., 2010) and LTP is impaired following inescapable, but not escapable, shock in a shuttle box avoidance task (Shors et al., 1989).

In summary, when an individual is subjected to environmental stressors in any phase of life, sleep is affected. Sleep disturbances after stress are modulated by several factors among which are the brain areas activated by stress, the ability to recover from stress, the behavioral coping strategy and ability to control the stressor.

## Clinical perspectives and closing comments

Sleep loss, sleep restriction, and the experience of being stressed are common place in our modern society. There is a broad consensus that insufficient sleep leads to a general slowing of response speed and increased variability in performance (Van Dongen et al., 2003). Whether sleep loss affects all cognitive processes and capacities, or specifically impairs some aspects of alertness, memory, perception and executive functions is a subject of debate (Killgore, 2010). Mood is especially sensitive to sleep loss. Chronic sleep disturbances are risk factors for developing anxiety and depression (Neckelmann et al., 2007), and vice versa, sleep disturbances are so frequently observed in patients experiencing psychological disorders that they form part of the diagnostic manual criteria for the disorders. Clinical studies of anxiety and depression indicate prevalence of both insomnia and hypersomnia (Ford and Kamerow, 1989; Ohayon, 2002; Riemann, 2007).

Modulation of recovery processes and neuroplasticity after brain trauma is sensitive to sleep loss. Insufficient sleep may compromise neuronal function and contribute to neurodegenerative processes. Disturbed sleep 3 days after focal cerebral ischemia is shown to reduce axonal sprouting, expression of synaptophysin, and the ischemia-stimulated neural and vascular cell proliferation in rats (Zunzunegui et al., 2011). The data suggests a role of sleep in the modulation of recovery processes and neuroplasticity after traumatic brain injury.

As the cell biological regulation of synaptic plasticity during sleep comes into view, new fundamental insights are likely to be gained regarding how information is processed and stored during the sleep cycle. Convergent evidence from electrophysiological, molecular, and behavioral studies all point to the importance of cyclic, synergistic interactions between NREM and REM stages in fulfilling the cognitive functions of sleep. Stress, sleep quality, and cognitive performance are inexorably intertwined. As reviewed here, differential effects of stressors on sleep quality, synaptic plasticity, and molecular mechanisms associated with synaptic plasticity have been established. A major challenge is to determine how different forms of stress (acute and chronic, controllable and uncontrollable) specifically alter the sleep cycle and the quality of the interactions between NREM and REM sleep. And reciprocally, how altered sleep habits may predispose to stress and maladaptive cognitive responses. More studies are needed to identify the specific neural circuits mediating stress-sleep interactions.

## Conflict of interest statement

The authors declare that the research was conducted in the absence of any commercial or financial relationships that could be construed as a potential conflict of interest.
